# Circulation of genotypes of dengue virus serotype 2 in Guangzhou over a period of 20 years

**DOI:** 10.1186/s12985-022-01773-7

**Published:** 2022-03-18

**Authors:** Liyun Jiang, Yuan Liu, Wenzhe Su, Yimin Cao, Qinlong Jing, Xinwei Wu, Zhicong Yang

**Affiliations:** 1 Virology Department, Guangzhou Centre for Disease Control and Prevention, Baiyunqu Qidelu 1, Guangdong, 510440 China; 2 Pestcide and Disinfection Department, Guangzhou Centre for Disease Control and Prevention, Baiyunqu Qidelu 1, Guangdong, 510440 China; 3 Epidemiology Department, Guangzhou Centre for Disease Control and Prevention, Baiyunqu Qidelu 1, Guangdong, 510440 China; 4 Microbiology Department, Guangzhou Centre for Disease Control and Prevention, Baiyunqu Qidelu 1, Guangdong, 510440 China

**Keywords:** Dengue virus serotype 2, Phylogeny, Molecular evolution, Epidemiology

## Abstract

**Background:**

The dengue epidemic in Guangzhou has imposed a rising burden on society and health infrastructure. Here, we present the genotype data for dengue virus serotype 2 (DENV-2) to improve understanding of this dengue epidemic.

**Methods:**

We sequenced the envelope gene of DENV-2 obtained from patient serum samples and subsequently performed maximum-likelihood phylogenetic analysis using PhyMLv3.1, maximum clade credibility analysis using BEAST v.1.10.4, and selection pressure analysis using Datamonkey 2.0.

**Results:**

The prevalent DENV-2 strains identified in Guangzhou region are related to those in Southeast Asian countries. In particular, the Malaysia/Indian subcontinent genotype is prevailing in Guangzhou with no apparent genotype shift having occurred over the past 20 years. However, episodic positive selection was detected at one site.

**Conclusions:**

Local control of the DENV-2 epidemic in Guangzhou requires effective measures to prevent and monitor imported cases. Moreover, the shift between the Malaysia/Indian subcontinent genotype lineages, which originated at different time points, may account for the rise in DENV-2 cases in Guangzhou. Meanwhile, the low rate of dengue haemorrhagic fever in Guangzhou may be explained by the dominance of the less virulent Malaysia/Indian subcontinent genotype.

## Background

Dengue is caused by infection with dengue virus (DENV), which is a member of the genus *Flavivirus*, family *Flaviviridae*. Approximately 96 million dengue infections were estimated globally in 2010, of which 70% were in Asia [1]. The clinical manifestations of dengue range from mild fever, known as dengue fever, to lethal forms of dengue haemorrhagic fever (DHF) and dengue shock syndrome (DSS). No effective vaccines or drugs against DENV have been developed to date, despite some vaccines available are important to decreases hospitalization for severe cases. Therefore, prevention strategies, such as avoiding additional mosquito bites, cleaning vector breeding sites, and use of pesticides, are the primary methods employed to control dengue infection.

There are four serotypes of DENV, namely, dengue virus serotype 1–4 (DENV-1–4). Each serotype can be farther divided into several genotypes based on phylogenetic analysis. The envelope of DENV plays a significant role during infection. DENV particles bind to host cell through envelope receptors. However, this attachment can be prevented via binding of antibodies specific for the envelope protein. Meanwhile, to avoiding antibody neutralisation, the *env* gene exhibits a high mutation rate. Indeed, the *env* gene is widely used to classify viruses into genetic groups (“genotypes”) within serotypes [2].

Guangzhou, located at 113°E, 23°N, is an important external city of China. With a population of 12 million, Guangzhou has borne a heavy burden of dengue for nearly 40 years since it was first recorded in 1978. In fact, 59,334 dengue cases were reported in mainland China from 2005 to 2015 [3], of which 39,325 cases were reported in Guangzhou (66.28%).

Over the past 2 decades, the dengue epidemic in Guangzhou was dominated by dengue virus serotype 1 (DENV-1) [4, 5]. However, DENV-2 has also been detected and associated with outbreaks in various communities. DENV-2 reportedly exhibits greater potential to cause DHF/DSS and is more readily transmitted than the other three serotypes. In fact, secondary infection with DENV-2 after the first heterotypic infection is more likely to result in DHF/DSS than secondary infection of the other three serotypes [6]. Therefore, with the prevalence of DENV-1in Guangzhou, the spread of DENV-2 may increase the risk of DHF/DSS outbreaks. Furthermore, a specific genotype may have the propensity to cause DHF/DSS and be transmitted efficiently by vectors [7, 8]. Therefore, it is necessary to closely monitor the genetic diversity and genotype variations of DENV-2 to better understand the dengue epidemic in Guangzhou.

To this end, we analysed serum samples of patients suspected of being infected with dengue that had been sent to the Guangzhou Center for Disease Control and Prevention from 2001 to 2020. We believe that phylogenetic analysis of the DENV-2 strains present throughout Guangzhou during this time period will provide critical insights to scientists and public health officials tasked with prevention and control of dengue.

## Methods

### Sample collection

Serum samples were obtained from suspected patients presenting with symptoms suggestive of dengue, such as sudden high fever with headache, arthralgia, and/or myalgia, according to *diagnosis for dengue fever* promulgated by the National Health Commission of the People’s Republic of China [9].

### Virus isolation and sequencing

The serum samples were analysed by reverse transcription-quantitative polymerase chain reaction (RT-qPCR) using a dengue virus RT-qPCR kit (Jiangsu BioPerfectus Technologies Co., Ltd., China).

Samples from 2001to 2018 that tested positive by RT-qPCR were diluted 1:50 in RPMI-1640 medium (Life Technologies Corporation, Grand Island, NY, USA). *Aedes albopictus* cloneC6/36(ATCC CRL-1660) cell monolayers (Cell Bank of the Chinese Academy of Sciences, Shanghai, China) were inoculated with the diluted samples and incubated at 28 °C. Supernatants of cultures with cytopathic effects (CPE) observed within 7 days were harvested for further sequencing. Supernatants of cell cultures with no CPE were added to new C6/36 monolayers. Inoculated cells without CPE after three generations were considered negative for DENV isolation.

In 2019 and 2020, to improve sequence quality and fidelity, RNA extracted from serum samples was analysed via RT-qPCR and direct sequencing of the envelope gene without virus isolation, as recommended by Leitmeyer et al. [10]. Therefore, more sequences were acquired in 2019 than in other years.

Viral RNA was extracted from the supernatants of cell cultures (2001–2018) or sera of patients (2019–2020) using the QIAamp Viral RNA Mini kit (Qiagen, Germany) according to the manufacturer’s instructions. Nucleotides from positions 937 to 2376 of the DENV-2 coding envelope gene were amplified using the SuperScript III One-Step RT–PCR System with Platinum Taq DNA polymerase (Invitrogen, USA). The sense primer was 5ʹ-CCAGGCTTTACCATAATGGC-3ʹ and the anti-sense primer was 5ʹ-CCAGCTGCACAACGCAACCAC-3ʹ. The reaction was initiated at 50 °C for 30 min, followed by denaturation at 94 °C for 2 min; 40 cycles of denaturation at 94 °C for 30 s, primer annealing at 52 °C for 30 s, primer extension at 72 °C for 2 min, and a final extension step at 72 °C for 7 min. The product was sequenced using Sanger sequencing.

### Phylogenetic and molecular clock analysis

The maximum-likelihood phylogenetic tree of the obtained envelope sequences acquired in Guangzhou, along with the reference sequences retrieved from GenBank, was constructed using PhyML v3.1 [11]. The substitution model was determined by SMART model selection of the Bayesian information criterion [12]. The fast likelihood-based method of eBayes was applied owing to the large number of sequences. Genotypes were grouped according to the criteria defined by Rico-Hesse [2].

A maximum clade credibility (MCC) tree was constructed for the same sequences using BEAST v.1.10.4. The detected date for each sequence was used as the calibration date. The tree was visualised using Bayesian Evolutionary Analysis Utility (BEAUti) with the following settings: substitution model, GTR; base frequencies, estimated; site heterogeneity model, gamma + invariant sites; number of gamma categories, 4; clock type, uncorrelated relaxed clock; tree model, random starting tree; length of chain, 100,000,000; echo state to screen every 10,000; and log parameters every 10,000. The results of both the maximum-likelihood and MCC phylogenetic trees were visualised and edited using FigTree.

### Selection pressure analysis

Episodic adaptive selection was evaluated using the Mixed Effects Model of Evolution (MEME) algorithm implemented by Datamonkey 2.0 [13]. A positive selection signature for each site was determined when the β^+^ parameter, representing the rate of nonsynonymous substitutions (dN), was greater than α, representing the rate of synonymous substitutions (dS).

## Results

A total of 37,431 serum samples from suspected patients from 2001 to 2020 were sent to the lab at the Guangzhou Center for Disease Control and Prevention for RT-qPCR screening. A total of 2942 positive samples were identified. From these samples, 1003 sequences were obtained, comprising 754 DENV-1, 148 DENV-2, 63 DENV-3, and 38 DENV-4. Table 1 summarises the DENV-2 cases detected by RT-qPCR in the serum samples of patients suspected to have dengue from 2001 to 2020. There were 416 cases of DENV-2 infection, comprising 373 domestic cases (89.66%) and 43 (10.34%) imported cases. Cases from Southeast Asian countries constituted 83.72% (n = 36) of imported cases. Before 2010, only one DENV-2 infection was detected in 2005. Since 2010, DENV-2 infections have been detected each year.

**Table 1:** Annual domestic and imported DENV-2 cases using RT-qPCR for 2001–2020 serum samples in Guangzhou

Year	2005	2010	2011	2012	2013	2014	2015	2016	2017	2018	2019	2020	total
DENV2	1	2	2	2	2	10	21	37	48	192	96	3	416
Domestic cases (percentage)	0	2 (100%)	0	0	2 (100%)	9 (90%)	17 (80.95%)	30 (81.08%)	41 (85.42%)	183 (95.31%)	86 (89.58%)	2 (66.67%)	372 (89.42%)
Imported cases (percentage)	1 (100%)	0	2 (100%)	2 (100%)	0	1 (10%)	4 (19.05%)	7 (18.92%)	7 (14.58%)	9 (4.69%)	10 (10.42%)	1 (33.33%)	44 (10.58%)
Bangladesh	0	0	0	0	0	0	0	1	1	0	0	0	0
Angola	0	0	1	0	0	1	2	3	1	2	3	0	0
Cambodia	0	0	1	0	0	0	0	0	2	0	0	0	0
Fiji	0	0	0	1	0	0	0	0	0	0	0	0	0
India	0	0	0	1	0	0	1	0	0	0	0	0	0
Indonesia	1	0	0	0	0	0	1	1	0	0	2	0	0
Malaysia	0	0	0	0	0	0	0	1	1	4	4	1	0
Maldives	0	0	0	0	0	0	0	1	0	0	0	0	0
Myanmar	0	0	0	0	0	0	0	0	2	0	0	0	0
Solomon Islands	0	0	0	0	0	0	0	0	0	1	0	0	0
Sri Lanka	0	0	0	0	0	0	0	0	0	1	0	0	0
Thailand	0	0	0	0	0	0	0	0	0	1	0	0	0
Vietnam	0	0	0	0	0	0	0	0	0	0	1	0	0

**Fig. 1:**
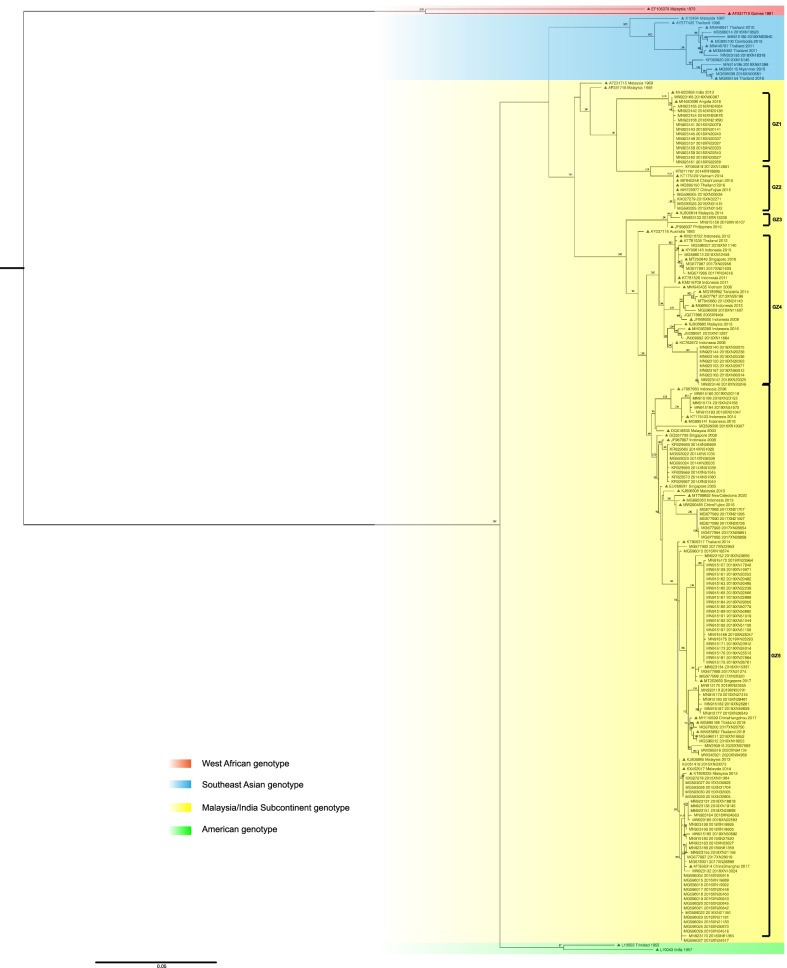
Maximum-likelihood midpoint rooted phylogenetic tree of DENV-2 envelope sequences from Guangzhou and reference sequences. Guangzhou sequences (n = 148) are identified according to the accession number, isolated year, and lab number. Reference sequences (n = 56) from GenBank (marked with a triangle) are identified using the accession number, country, and year. Bootstrap support, with > 75%, is shown next to the branches. Different colours present different genotypes

Over the 20 years, 148 DENV-2 envelope gene sequences (1440 bp) were obtained from isolated strains or sera of patients in Guangzhou. All sequences were deposited in GenBank. The phylogenetic tree shown in Fig. 1 was constructed based on these 148 sequences detected in Guangzhou and 56 sequences downloaded from GenBank. Most of the sequences (n = 142, 95.95%) identified from 2001 to 2020 in Guangzhou belonged to the Malaysia/Indian subcontinent genotype, while only six sequences (4.05%) clustered in the Southeast Asia genotype. No American genotype or West African genotype was detected throughout the study period.

The Malaysia/Indian subcontinent genotype can be further divided into several lineages: GZ1, GZ2, GZ3, GZ4, and GZ5. The sequences acquired in 2005, 2010, and 2013 all belonged to lineage GZ4; thereafter, a shift was observed to lineage GZ5 in 2014. Moreover, nine subspecies were detected in 2015, three of which (33.33%) clustered in lineage GZ2, while the remaining six (66.67%) clustered in lineage GZ5. Thereafter, most sequences belonged to lineage GZ5; however, a few were scattered among the GZ1, GZ2, GZ3, and GZ4 lineages.

**Fig. 2:**
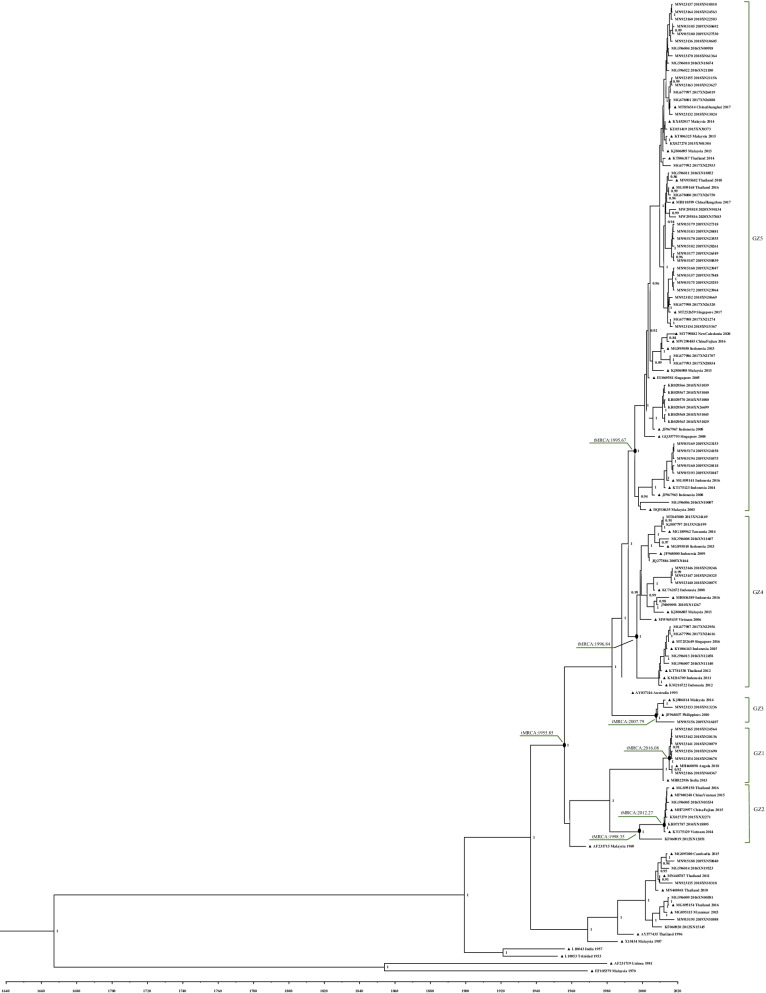
Maximum clade credibility tree of envelope gene nucleotide sequences collected in Guangzhou and from GenBank. Guangzhou sequences, n = 80; GenBank sequences (triangle), n = 53. Posterior probabilities higher than 0.80 are shown at each node. tMRCA = time to the most recent common ancestor

When several identical sequences were detected in the same year, one was retained as a representative for constructing the MCC tree (Fig. 2). This tree was based on 80 sequences obtained in Guangzhou along with 53 sequences retrieved from GenBank. All of the Malaysia/Indian subcontinent genotype strains shared an ancestor in 1955. However, the various lineages of this genotype manifested different introduction times. Lineage GZ5, which was the most prevalent, emerged in 1995 and comprised sequences from 2014 to 2019.

Positive selection of the envelope gene for lineage GZ5was analysed using MEME. The sequences of Guangzhou were compared with those of the reference sequence DQ518635 isolated from Malaysia in 2003. Episodic positive selection was detected at one site, codon position 364, with β^+^ = 2160.36, α = 0.00, *P* = 0.06. Additionally, molecular characterisation identified two amino acid differences (E329D and I439V) in all sequences from the Guangzhou isolates in comparison with the DQ518635 sequence from Malaysia in 2003.

## Discussion

Over the last century, DENV-2 epidemics were reported in 1986, 1987, 1988, and 1993 in Guangzhou [14, 15], thereafter, they have subsided. However, our findings indicate that DENV-2 has been spreading throughout Guangzhou from 2001 to 2020. Although no DENV-2 infection was detected prior to 2005, the number of annual DENV-2 cases continued to increase between 2010 and 2018. We also determined that the percentage of DENV-2 domestic cases increased from 80.95 to 95.31% between 2015 and 2018, reaching a peak in both number and percentage in 2018. Thereafter, the number and percentage of domestic cases declined slightly in 2019 but remained the second highest compared to those in previous years. Moreover, a sharp decrease in DENV-2 cases occurred in 2020, which might be related to the quarantine imposed due to the coronavirus disease 2019 outbreak [16]. That is, limiting imported cases with the quarantine may have controlled the local dengue epidemic, highlighting the relevance of monitoring imported cases for local control.

The DENV-2 epidemic in Guangzhou is greatly impacted by Southeast Asian countries. In fact, our epidemiological investigation revealed that 83.72% of DENV-2 imported cases, most of which were returning travellers, originated from Southeast Asian countries. The results of a BLAST search in GenBank and the phylogenetic tree (Fig. 1) further revealed that the DENV-2 strains sequenced in Guangzhou were closely related to those in Southeast Asian countries, which was similar to the characteristic of dengue epidemics involving the other three serotypes in Guangzhou [4, 17, 18]. Indeed, the World Health Organization statistics revealed that infections in Southeast Asian countries account for half of the global dengue burden. Meanwhile, from 2015 to 2019, dengue cases in Southeast Asia increased by 46% (from 451,442 to 658,301) [19]. China not only contiguous with Southeast Asian countries, but also is farther connected by economic ties. Moreover, with the opening of private travel abroad, the number of travellers to Southeast Asian countries has steadily increased [20–22]. This situation is not unique to China, as the spread of dengue viruses by travellers has become a global issue [23]. Therefore, better preparation is needed, with strict regulations, to prevent the spread of infection when travelling in endemic areas. For example, a convenient and rapid method for screening viremia that can be applied at customs may help curb the import and spread of DENV.

The Malaysia/Indian subcontinent genotype was responsible for the epidemic in Guangzhou. As displayed in the phylogenetic tree in Fig. 1, this genotype, shown in yellow, constitutes 95.95% (142 sequences) of the total 148 DENV-2 sequences. Meanwhile only six sequences of the Southeast Asia genotype were detected. Moreover, no genotype shift in the Malaysia/Indian subcontinent genotype was observed over the 20-year study period. The most recent common ancestor of all Malaysia/Indian subcontinent strains was estimated to have appeared in 1955. However, when dividing the Malaysia/Indian subcontinent genotype into its different lineages, a shift from lineage GZ4 to GZ5, was observed between 2013 and 2014, which coincided with a rising number of DENV-2 cases since 2014. Although there is no clear relationship between lineage and virulence in DENV, outbreaks, limited circulation, and spreading related to shifts in lineages have been reported [24–27]. Substitutions in the envelope gene that may result in maturation and activation of macrophages, with consequent enhancement of the immune response characterised by increased production of cytokines, are considered to be likely causes of the prevailing differences among lineages. Meanwhile, positive selection analysis of the GZ5 lineage by MEME exhibited signs of directional selection, which may contribute to the prevalence of GZ5. However, further research is needed to confirm whether this lineage shift is responsible for the rise in cases.

With the prevalence of the Malaysia/Indian subcontinent genotype in Guangzhou, the relatively low rate of DHF/DSS suggests that this genotype may be less virulent. Secondary infection with DENV-2 after infection with heterotypic DENV is believed to be associated with an increased risk of DHF/DSS [6]. However, with the DENV-1epidemic in Guangzhou persisting for more than 20 years and the rising number of DENV-2 cases [4, 5], the incidence of DHF/DSS remained relatively low compared with the global incidence and that of Southeast Asian countries [28–31]. Another study revealed that secondary infection with the American genotype of DENV-2 failed to cause DHF/DSS [32], whereas other extensive studies indicated that the Southeast Asian genotype was more efficient at infection and was also more likely to cause DHF/DSS [7, 8, 32, 33]. Of the 148 sequences detected in Guangzhou, only six (4.05%) belonged to the Southeast Asia genotype, four of which were identified from imported cases. This revealed that the Southeast Asia genotype was rare in Guangzhou, which may explain the low incidence of DHF/DSS. Meanwhile, the prevailing Malaysia/Indian subcontinent genotype has a limited capacity for leading to DHF/DSS. However, further studies are needed to determine whether the incidence of DHF/DSS is low in other areas, with an epidemic dominated by the Malaysia/Indian subcontinent genotype. Determining the critical differences between genotypes and host immune mechanisms that may enhance the pathogenesis of genotypes might provide new insights to advance the current understanding regarding the mechanism of DHF/DSS.

The DENV-2 epidemic in Guangzhou was complex. Specifically, the MCC tree in Fig. 2 revealed that the Guangzhou strains originated from different time periods, indicating various evolution and dissemination paths. Strains that clustered into lineage GZ5 shared the eldest ancestor in 1995, whereas strains belonging to lineage GZ1 emerged in 2016. Meanwhile, the domestic and foreign strains of lineage GZ2 detected in 2014 and 2016 shared the same ancestor in 2012. The strain then evolved during 2014 and 2016 in Guangzhou and spread not only in China but also throughout Thailand and Vietnam. However, within the same year, the strains may have also be distributed in different lineages; for example, the sequences from isolates obtained in 2018 were distributed among lineages GZ1, GZ3, GZ4 and GZ5. This co-epidemic of different lineages showing different origins has complicated the epidemic situation in Guangzhou.

## Conclusions

Our study has investigated the epidemiology of DENV-2 and shed light on epidemiologic impact of the Malaysia/Indian subcontinent genotype and its different lineages, in Guangzhou, an important dengue circulation city in China. In particular, the increase in DENV-2 cases may be due to an observed lineage shift. Taken together, our study findings suggest that global cooperation is required to curb the spread of dengue.

## Data Availability

The sequences analysed during the current study are available in the GenBank repository, https://www.ncbi.nlm.nih.gov/genbank/. The data that support the findings of this study are available from the corresponding author.

## Abbreviations

BEAUti: Bayesian Evolutionary Analysis Utility
CPE: Cytopathic effects
DENV: Dengue virus
DENV1-4: Dengue virus serotype 1–4
DHF: Dengue haemorrhagic fever
DSS: Dengue shock syndrome
MCC: Maximum clade credibility
MEME: Mixed Effects Model of Evolution
